# Driven translocation of a semi-flexible polymer through a nanopore

**DOI:** 10.1038/s41598-017-07227-3

**Published:** 2017-08-07

**Authors:** Jalal Sarabadani, Timo Ikonen, Harri Mökkönen, Tapio Ala-Nissila, Spencer Carson, Meni Wanunu

**Affiliations:** 1 Department of Applied Physics and COMP Center of Excellence, Aalto University School of Science, P.O. Box 11000, FI-00076 Aalto, Espoo Finland; 2 VTT Technical Research Centre of Finland Ltd., P.O. Box 1000, FI-02044 VTT, Finland; 3 Department of Mathematical Sciences and Department of Physics, Loughborough University, Loughborough, Leicestershire LE11 3TU UK; 4 Department of Physics, Northeastern University, Boston, MA 02115 United States

## Abstract

We study the driven translocation of a semi-flexible polymer through a nanopore by means of a modified version of the iso-flux tension propagation theory, and extensive molecular dynamics (MD) simulations. We show that in contrast to fully flexible chains, for semi-flexible polymers with a finite persistence length ${\tilde{{\boldsymbol{\ell }}}}_{{\boldsymbol{p}}}$ the *trans* side friction must be explicitly taken into account to properly describe the translocation process. In addition, the scaling of the end-to-end distance *R*_N_ as a function of the chain length N must be known. To this end, we first derive a semi-analytic scaling form for *R*_N_, which reproduces the limits of a rod, an ideal chain, and an excluded volume chain in the appropriate limits. We then quantitatively characterize the nature of the trans side friction based on MD simulations. Augmented with these two factors, the theory shows that there are three main regimes for the scaling of the average translocation time *τ* ∝ *N* ^*α*^. In the rod ${\boldsymbol{N}}{\boldsymbol{/}}{\tilde{{\boldsymbol{\ell }}}}_{{\boldsymbol{p}}}{\boldsymbol{\ll }}1$, Gaussian ${\boldsymbol{N}}{\boldsymbol{/}}{\tilde{{\boldsymbol{\ell }}}}_{{\boldsymbol{p}}}\sim {\bf{1}}{{\bf{0}}}^{{\bf{2}}}$ and excluded volume chain ${\boldsymbol{N}}{\boldsymbol{/}}{\tilde{{\boldsymbol{\kappa }}}}_{{\boldsymbol{p}}}$ ≫ **10** ^**6**^ limits, *α* = 2, 3/2 and 1 + *ν*, respectively, where ν is the Flory exponent. Our results are in good agreement with available simulations and experimental data.

## Introduction

Since the seminal works by Bezrukov *et al*.^1^ in 1994, and two years later by Kasianowicz *et al*.^2^, polymer translocation through nanopores has become one of the most active research topics in soft condensed matter physics^3–5^. It plays an important role in many biological processes such as virus injection and protein transportation through membrane channels^6^. It also has many technological applications such as drug delivery^7^, gene therapy and rapid DNA sequencing^2, 8–11^, and has been motivation for many experimental and theoretical studies^3–5, 12–47^.

**Figure 1:**
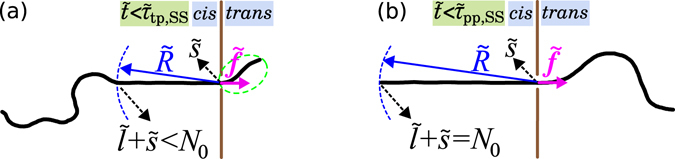
(**a**) A schematic of the translocation process in the tension propagation (TP) stage, i.e. $\tilde{t} < {\tilde{t}}_{tp,SS}$, for the strong stretching (SS) regime. *N*_0_ is the contour length of polymer and the translocation coordinate $\tilde{s}$ equals the number of beads that have already been translocated into the *trans* side. The number of beads influenced by the tension force is $\tilde{l}+\tilde{s}$, which during TP stage is less than *N*_0_. $\tilde{R}$ determines the location of the tension front. (**b**) The translocation process for SS regime during the post propagation stage where the tension front has reached the chain end, which yields $\tilde{l}+\tilde{s}={N}_{0}$. The *cis* and the *trans* sides denote the regions where the translocation process starts and where the chain translocates, respectively, as indicated in the figure.

Most analytical and theoretical studies to date have focused on field-driven translocation of flexible polymers through nanopores. A break-through in this problem came from Sakaue, who employed the idea of *tension propagation* (TP) to explain the physical mechanism of the driven translocation process^21^. According to TP theory when the external driving force, which is due to an external electric field across the pore, acts on the bead(s) at the pore in the direction of *cis* to *trans* side (see Fig. 1), a tension front propagates along the backbone of the chain in the *cis* side of the chain. Consequently, the *cis* side is divided into mobile and immobile parts, where the mobile part of the chain has been already influenced by the tension force and moves towards the pore, and the immobile part of the chain is in its equilibrium state, i.e. its average velocity is zero.

Following Sakaue’s work, in a series of papers Ikonen *et al*. developed a Brownian dynamics - TP theory (BDTP) to take into account the effect of pore friction, finite chain length, and thermal fluctuations due to the solvent during the course of translocation^30, 31^. Most recently, the BDTP theory was reformulated within the constant monomer *iso-flux* approximation^25^ (IFTP)^32, 33^, leading to a fully quantitative and self-consistent theory of dynamics of driven translocation with only one free parameter, the effective pore friction. A key role in the theory is played by the total effective friction, which comprises the constant pore friction (interaction of the monomers with the nanopore) and drag from the *cis* part of the chain. For fully flexible chains, the contribution from the *trans* side of the friction can be included in the pore friction, and need not be explicitly considered.

However, in many cases of practical interest the translocating polymers are not fully flexible–e.g. for double-stranded DNA, the persistence length ${\ell }_{p}$ is typically about 500 Å. To unravel the influence of stiffness to translocation, in this paper we consider the pore-driven translocation dynamics of semi-flexible polymers with a finite persistence length within the IFTP theory. We argue that unlike for fully flexible chains, the *trans* side friction has a significant contribution to the dynamics and must be explicitly added to the expression for the total friction. To calculate the chain drag, we derive a semi-analytic form for the end-to-end scaling form *R*_*N*_ for semi-flexible chains, which correctly incorporates the various scaling regimes and crossover between them for different ratios of the persistence and chain lengths ${\tilde{\ell }}_{p}/N$. Neither of these factors have been considered in the previous works^39–42^. When properly augmented with the correct end-to-end scaling form and time-dependent *trans* side friction, the IFTP theory shows that the average translocation time displays complex scaling and crossover behavior as a function of ${\mathop{\ell }\limits^{ \sim }}_{p}/N$. In the appropriate limits, the IFTP theory also recovers the exactly known results for the scaling exponent of the translocation time. It is important to note that in the IFTP theory there is only one unknown parameter, the *effective pore friction η*_p_, which can be obtained either experimentally or from MD simulations^30–33^.

## Results

### Theory

- Strong stretching regime.

In the IFTP theory, we use dimensionless units denoted by tilde as $\tilde{X}\equiv X/{X}_{u}$, with the units of length *s*_*u*_ ≡ *σ*, time *t*_*u*_ ≡ *ησ* ^2^/(*k*_*B*_ *T*), force *f*_*u*_ ≡ *k*_*B*_ *T*/*σ*, velocity *v*_*u*_ ≡ *σ*/*t*_*u*_ = *k*_*B*_ *T*/(*ησ*), friction Γ_*u*_ ≡ *η*, and monomer flux *ϕ*_*u*_ ≡ *k*_*B*_ *T*/(*ησ* ^2^), where *σ* is the segment length, *T* is the temperature of the system, *k*_*B*_ is the Boltzmann constant, and *η* is the solvent friction per monomer. The quantities without the tilde, such as the force, friction and length, are expressed in Lennard-Jones units.

In the SS regime, where it is sufficient to use the deterministic limit of the IFTP theory^30–33^, the equation of motion for the translocation coordinate $\tilde{s}$ which is the number of beads in the *trans* side (see Fig. 1), is given by

1 $$\tilde{{\rm{\Gamma }}}(\tilde{t})\frac{d\tilde{s}}{d\tilde{t}}=\tilde{f},$$

where $\tilde{{\rm{\Gamma }}}(\tilde{t})$ is the effective friction and $\tilde{f}$ is the external driving force.

In the iso-flux assumption the monomers flux, $\tilde{\varphi }\equiv d\tilde{s}/d\tilde{t}$, on the mobile domain in the *cis* side and also through the pore is constant in space, but evolves in time^25, 32^. The tension front, which is the boundary between the mobile and immobile domains, is located at distance $\tilde{x}=-\tilde{R}(\tilde{t})$ from the pore. The external driving force acts on the monomer(s) inside the pore located at $\tilde{x}=0$ (see Fig. 1(a)).

It has been shown^30–34^ that for flexible polymers the friction can be written as $\tilde{{\rm{\Gamma }}}(\tilde{t})={\tilde{\eta }}_{{\rm{cis}}}(\tilde{t})+{\tilde{\eta }}_{{\rm{p}}}$, and the translocation dynamics is essentially controlled by the time-dependent friction ${\tilde{\eta }}_{{\rm{cis}}}(\tilde{t})$ on the *cis* side of the chain, whereas the *trans* side friction is negligible and can be absorbed into the constant pore friction ${\tilde{\eta }}_{{\rm{p}}}$. In the case of semi-flexible chains this approximation is not justified. Within the IFTP theory, the friction due to the *trans* side of the chain ${\tilde{\eta }}_{{\rm{TS}}}$ can be taken into account as follows. In the strong stretching (SS) regime of strong driving, where the mobile part of the chain in the *cis* side is fully straightened (cf. Fig. 1(a) and (b)), we can integrate the force balance equation over the mobile domain^32^ and the monomer flux becomes

2 $$\tilde{\varphi }(\tilde{t})=\frac{\tilde{f}}{\tilde{R}(\tilde{t})+{\tilde{\eta }}_{{\rm{p}}}+{\tilde{\eta }}_{{\rm{TS}}}}\mathrm{.}$$

By combining Eqs (1) and (2), the effective friction is obtained as

3 $$\tilde{{\rm{\Gamma }}}(\tilde{t})=\tilde{R}(\tilde{t})+{\tilde{\eta }}_{{\rm{p}}}+{\tilde{\eta }}_{{\rm{TS}}}\mathrm{.}$$

The time evolution of $\tilde{s}$ is determined by Eqs (1), (2) and (3), but knowledge of the position of the tension front on the *cis* side of the chain $\tilde{R}(\tilde{t})$ is still required to find the full solution. We will derive the equation of motion for $\tilde{R}(\tilde{t})$ separately for the TP and *post propagation* (PP) stages. In the TP stage the tension has not be reached the chain end as presented in Fig. 1(a), while in the PP stage the final monomer has been already influenced by the tension force (see Fig. 1(b)).

**Figure 2:**
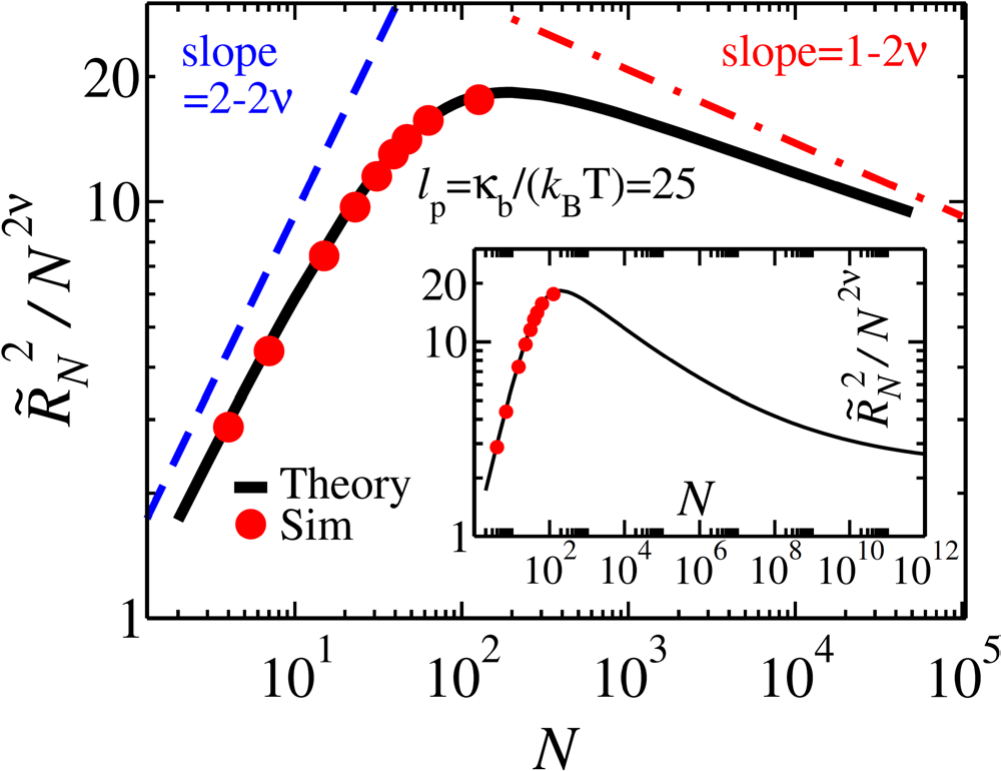
Normalized end-to-end distance ${\tilde{R}}_{N}^{2}/{N}^{2\nu }$ as a function of the contour length of the polymer *N* for fixed value of bending rigidity (in the MD simulations) *κ*_*b*_ = 30, which corresponds to ${\ell }_{p}=25$, when *k*_*B*_ *T* = 1.2. The black curve shows the analytical formula of Eq. (4) while red dots present the MD simulations results. Inset shows crossover from Gaussian to self-avoiding behavior for an extended range of *N*.
- Time evolution of the tension front.

- End-to-end distance of a semi-flexible chain.
  To find the equation of motion for $\tilde{R}(\tilde{t})$, which is the root-mean-square of the end-to-end distance, an analytical form of $\tilde{R}(\tilde{t})$ for semi-flexible chains is needed. To this end, we have carried out extensive MD simulations of bead-spring models of semi-flexible chains in 3D. The technical details can be found in the Supplementary. The MD simulations have been done for different values of contour length *Nσ* and bending rigidity *κ*_*b*_. In 3D the persistence length ${\ell }_{p}$ can be expressed as a function of *κ*_*b*_ as ${\ell }_{p}={\kappa }_{b}/({k}_{B}T)$. We find that the MD data (cf. Fig. 2) is well described by the following interpolation formula for the end-to-end distance of a semi-flexible chain:
  4 $${\tilde{R}}_{N}={\{{\tilde{R}}_{F}^{2}-\frac{{\tilde{R}}_{F}^{4}}{2{a}_{1}{N}^{2}}[1-\exp (-\frac{2{a}_{1}{N}^{2}}{{\tilde{R}}_{F}^{2}})]+2{\tilde{\ell }}_{p}N-\frac{2{\tilde{\ell }}_{p}^{2}}{{b}_{1}}[1-\exp (-\frac{{b}_{1}N}{{\tilde{\ell }}_{p}})]\}}^{\frac{1}{2}}\mathrm{.}$$
  Here ${\tilde{R}}_{F}=A{\tilde{\ell }}_{p}^{{\nu }_{p}}{N}^{\nu }$, with ν _p_ = 1/(*d* + 2) (*d* = 3) which describes the scaling of the chain in the limit $N/{\tilde{\ell }}_{p}$ ≫ 1^48^ and is correctly recovered by Eq. (4). In the opposite stiff or rod-like chain limit of $N/{\tilde{\ell }}_{p}$ ≪ 1, Eq. (4) recovers the trivial result that ${\tilde{R}}_{N}=N$. The quantity *ν* = 0.588 is the Flory exponent, and *A* = 0.8, *a*_1_ = 0.1 and *b*_1_ = 0.9 are constants. In the intermediate regime $4\lesssim N/{\tilde{\ell }}_{p}\lesssim 400$ which here corresponds to ${10}^{2}\lesssim N\lesssim {10}^{4}$ for ${\tilde{\ell }}_{p}=25$, a crossover occurs from a rod-like chain to a Gaussian (ideal) polymer, followed by an eventual crossover to a self-avoiding chain for $N/{\tilde{\ell }}_{p}$ ≫ 10^6^ ^49^ as can be seen in the inset of Fig. 2. It should be noted that the amplitude *A* is fixed by the equilibrium scaling of the chain, and thus only *a*_1_ and *b*_1_ are fitting parameters. We note that although Eq. (4) is reasonably accurate and valid with the same fixed values of *A*, *a*_1_ and *b*_1_ for a wide range of values of ${\tilde{\ell }}_{p}$, as shown in the Supplementary, it does not recover the result of the perturbation theory in the limit of small (but not negligible) excluded volume.

Using $\tilde{R}(\tilde{t})$ in Eq. (4) together with the mass conservation $N=\tilde{l}+\tilde{s}$, where $\tilde{l}\,=\,\tilde{R}$, the equation of motion for the tension front in the TP stage for the SS regime (see Fig. 1(a)) can be derived as

5 $$\dot{\tilde{R}}(\tilde{t})=\frac{\tilde{\varphi }(\tilde{t})({\mathcal{G}}+ {\mathcal H} )}{2\tilde{R}(\tilde{t})-({\mathcal{G}}+ {\mathcal H} )},$$

where

6 $$\begin{array}{rcl}{\mathcal{G}} & = & \frac{{\tilde{R}}_{F}^{2}}{N}[2\nu -(2-2\nu )\exp (-\frac{2{a}_{1}{N}^{2}}{{\tilde{R}}_{F}^{2}})]+\frac{\mathrm{(4}\nu -\mathrm{2)}{\tilde{R}}_{F}^{4}}{2{a}_{1}{N}^{3}}[-1+\exp (-\frac{2{a}_{1}{N}^{2}}{{\tilde{R}}_{F}^{2}})],\\  {\mathcal H}  & = & 2{\tilde{\ell }}_{p}[1-\exp (-\frac{{b}_{1}N}{{\tilde{\ell }}_{p}})]\mathrm{.}\end{array}$$

In the PP stage (see Fig. 1(b)) the correct closure relation is $\tilde{l}+\tilde{s}={N}_{0}$. Then one can derive the equation of motion for the tension front in PP stage as

7 $$\dot{\tilde{R}}(\tilde{t})=-\tilde{\varphi }(\tilde{t}\mathrm{).}$$

To find the solution, in the TP stage, Eqs (1), (2), (3) and (5) must be solved self-consistently while in the PP stage, Eqs (1), (2), (3), (7) must be solved.

### Trans side friction

In the Supplementary we present the waiting time distribution $w(\tilde{s})$, which is the time that each bead spends at the pore. The data clearly show that in order to have a quantitative theory, we must include ${\tilde{\eta }}_{{\rm{TS}}}(t)$ in Eq. (3).

**Figure 3:**
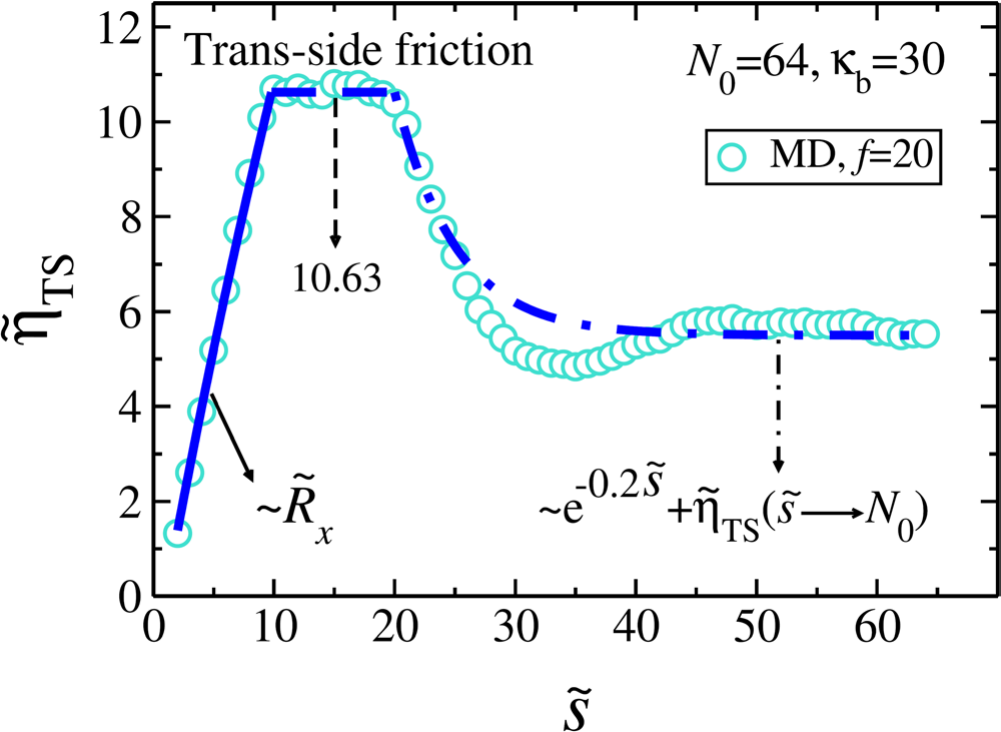
The *trans* side friction ${\tilde{\eta }}_{{\rm{TS}}}(\tilde{s})$ as a function of $\tilde{s}$ for fixed values of the chain length *N*_0_ = 64, bending rigidity *κ*_*b*_ = 30, and external driving force *f* = 20. The turquoise circles are MD data. The blue solid, dashed and dashed-dotted lines represent the three different regimes (see the text and the Supplementary for details).

It is expected that the *trans* side friction is a complicated function of the driving force, chain length and the bending rigidity, and the present IFTP theory does not allow us to derive it analytically. To this end, we have extracted it numerically from the MD simulations as shown in Fig. 3. Details and additional data for smaller driving forces and for different persistence lengths are in the Supplementary. We can identify three distinct regimes in ${\tilde{\eta }}_{{\rm{TS}}}(\tilde{s})$. For small $\tilde{s}/{N}_{0}$, we find that the friction grows proportional to the *x* component of the end-to-end distance ${\tilde{R}}_{x}$. After this initial stage it saturates to a constant value (here ≈ 10.63), which from the MD simulations indicates buckling of the *trans* part of the chain. This buckling of the chain reduces the friction and we find an approximately exponential decay of the friction towards an asymptotic constant ${\tilde{\eta }}_{{\rm{TS}}}({N}_{0})\approx 5.5$.

### Translocation time exponent

The scaling of the average translocation time as a function of the chain length $\tau \propto {N}_{0}^{\alpha }$ is a fundamental characteristic of translocation dynamics. For flexible chains it scales as $\tau ={a}_{p}{N}_{0}+{a}_{c}{N}_{0}^{\nu +1}$, where *a*_*p*_ and *a*_*c*_ are constants. The first term is due to the pore friction which causes a significant finite-size correction to the asymptotic scaling where *α* = *ν* + 1^30–34^. The asymptotics is, of course, recovered for the semi-flexible chains in the large *N*_0_ limit when ${\tilde{\ell }}_{p}/{N}_{0}\ll 1$. On the other hand, in the limit of a rod-like polymer $\tau \propto {N}_{0}^{2}$. Following ref. 32, we can derive an analytic expression for *τ*. Using Eq. (2) the total translocation time can be written as

8 $$\tilde{\tau }=\frac{1}{\tilde{f}}[{\int }_{0}^{{N}_{0}}{\tilde{R}}_{N}dN+{\tilde{\eta }}_{{\rm{p}}}{N}_{0}]+{\tilde{\tau }}_{{\rm{TS}}},$$

where ${\tilde{\tau }}_{{\rm{TS}}}=[{\int }_{0}^{{N}_{0}}{\tilde{\eta }}_{{\rm{TS}}}dN+{\int }_{0}^{{\tilde{R}}_{{N}_{0}}}({\tilde{\eta }}_{{\rm{TS}},{\rm{pp}}}-{\tilde{\eta }}_{{\rm{TS}},{\rm{tp}}})d\tilde{R}]/\tilde{f}$ is the *trans* side contribution to the translocation time. The second term in ${\tilde{\tau }}_{{\rm{TS}}}$ is due to non-monotonic behavior of ${\tilde{\eta }}_{{\rm{TS}}}$ in the TP and PP stages. In the rod limit we obtain the simple analytical result that

9 $$\tilde{\tau }=\frac{1}{\tilde{f}}[{\tilde{\eta }}_{p}{N}_{0}+{N}_{0}^{2}],$$

which gives the asymptotic exponent *α* = 2. The corresponding effective exponents will be between unity and two.

To quantify the influence of the *trans* side and pore friction on the effective translocation exponent we define two rescaled translocation exponents *α* ^†^ and *α* ^‡^ as ${\tau }^{\dagger }=\tau -{\tau }_{{\rm{TS}}} \sim {N}_{0}^{{\alpha }^{\dagger }}$ and ${\tau }^{\ddagger}=\tau -{\tau }_{TS}-{a}_{p}{N}_{0} \sim {N}_{0}^{{\alpha }^{\ddagger}}$, respectively. In the short (${N}_{0}/{\tilde{\ell }}_{p}\lesssim 4$) and intermediate ($4\lesssim {N}_{0}/{\tilde{\ell }}_{p}\lesssim 400$) chain limits, contributions from both the *trans* side and pore friction are important as can be seen in Eq. (8).

**Figure 4:**
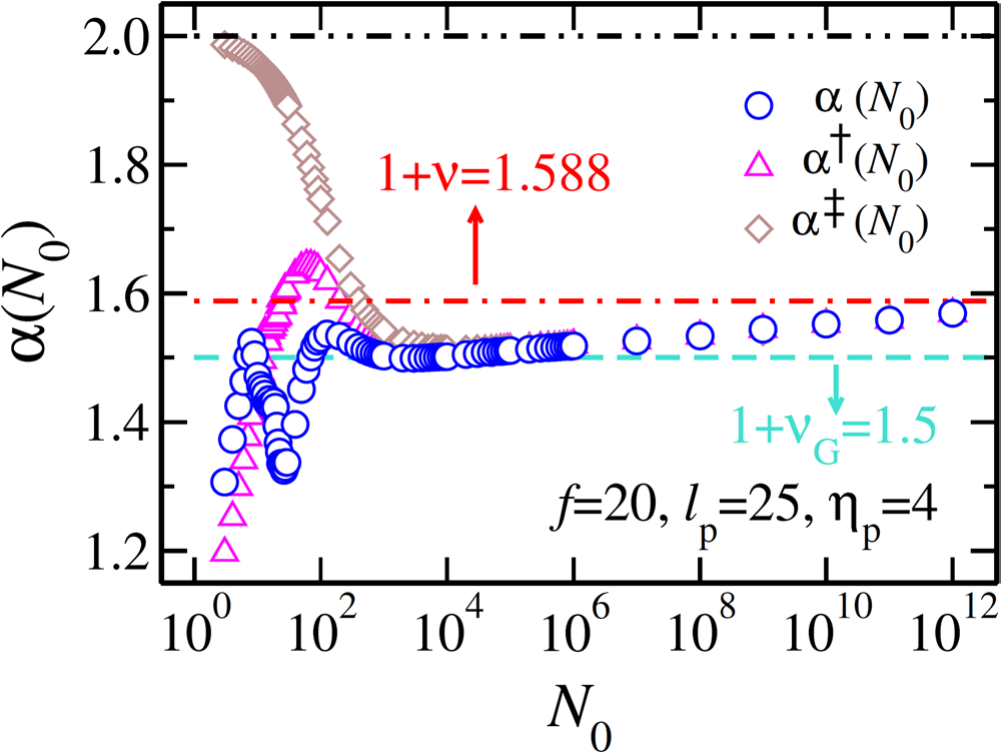
The effective translocation time exponents as a function of the chain length *N*_0_ for ${\ell }_{p}=25$ and pore friction *η*_p_ = 4, and external driving force *f* = 20. The blue circles show the translocation exponent *α* as a function of *N*_0_, while pink triangles and brown diamonds show the rescaled translocation exponents *α* ^†^ and *α* ^‡^, respectively. The horizontal black dashed-dotted-dotted, red dashed-dotted and turquoise dashed lines show the asymptotic rod-like, excluded volume chain and Gaussian scaling limits, respectively. See text for details.

In Fig. 4 we show the detailed dependence of the effective translocation time exponents as a function of the chain length *N*_0_ for constant values of the persistence length ${\ell }_{p}=25$, pore friction *η*_p_ = 4 and driving force *f* = 20. The blue circles show the effective value of the total *α* as a function of *N*_0_. The non-monotonic behavior of the *trans* side friction leads into a non-monotonic dependence of *α* on *N*_0_. Interestingly enough, there is an extended intermediate range of chain lengths where the exponent is very close to the Gaussian value *α* = 3/2 and slowly approaches its asymptotic value of 1 + *ν* = 1.588 from below. We note that in order to see this crossover it is necessary to have a full scaling form for the end-to-end distance of the form of Eq. (4).

To quantify how the *trans* side friction affects the effective translocation exponent, in Fig. 4 we plot *α* ^†^ (pink triangles). It approaches *α* for *N*_0_ > 10^4^, where the *trans* side friction becomes negligible. Finally, the rescaled translocation exponent *α* ^‡^ (brown diamonds), which is the effective translocation time exponent in the absence of both *trans* side and pore friction, is also plotted as a function of *N*_0_. This exponent recovers the rod-like limit for very short chains. It merges with the other two effective exponents to the almost Gaussian value at intermediate lengths and eventually approaches *ν* + 1, as expected.

Each data point in Fig. 4 for the effective exponents *α*, *α* ^†^ and *α* ^‡^, is numerically obtained by linear regression model from three consecutive [ln(*τ*), ln(*N*_0_)] data points.

**Figure 5:**
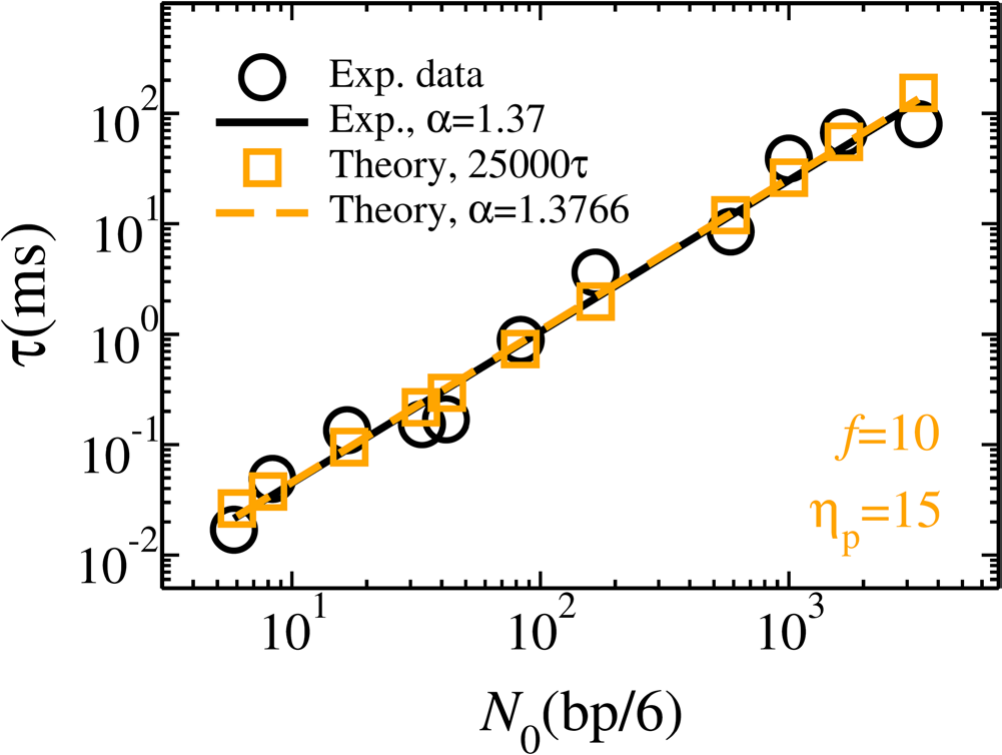
Translocation time *τ* as a function of the chain length *N*_0_. Black circles are experimental data in Fig. 6(c) of ref. 14 while orange squares are data from the IFTP theory, where we have used the coarse grained model. Each bead contains 6 bps. The value of the external driving force in the IFTP theory is *f* = 10 and the pore friction is *η*_p_ = 15. The translocation time for the IFTP theory has been multiplied by a factor of 25000 to agree with the experimental time scale. The black solid and orange dashed lines are linear fitting curves to experimental and IFTP theory, respectively. Similar results can be obtained for the values of the external driving forces *f* = 5 and 20. Details on mapping the experimental data to theory are explained in Sec. I of the Supplementary.

Finally, we compare the results of IFTP theory with relevant experiments. In Fig. 5, we present the translocation time obtained from experiments (black circles) and from the augmented IFTP theory (orange squares) as a function of the chain length *N*_0_(bp/6), for fixed values of external driving force *f* = 10 and pore friction *η*_p_ = 15. The value of external driving force *f* = 10 corresponds to potential difference *V* = 200 mV across the pore in the experiments^14^ (for more information see the Supplementary). This assumes a negligible field outside the pore which is predominantly due to access-resistance, in accordance with finite-element simulations^14^. To match the length scales, we coarse grain such that one bead in our model contains 6 bps. With this choice the translocation exponent from the IFTP theory (orange dashed line) is in good agreement with the exponent from the experimental data (black solid line).

## Discussion

We have shown here that in addition to the case of fully flexible polymers, the IFTP theory provides the proper theoretical framework for driven translocation of semi-flexible polymers. The two key quantities required are an explicit determination of the *trans* side friction and a proper analytical formula for the end-to-end distance of semi-flexible polymers. The augmented IFTP theory can quantitatively describe all the relevant scaling regimes for the scaling exponent of the average translocation time, and crossover between them. It also reproduces the exactly known limits and is in good agreement with available experimental data.

## Methods

### Molecular dynamics model

In our MD simulations the polymer is modeled by a bead-spring chain^50^. The excluded volume interaction between the beads is given by the repulsive Lennard-Jones (LJ) potential ${U}_{{\rm{LJ}}}=4\varepsilon [{(\frac{\sigma }{r})}^{12}-{(\frac{\sigma }{r})}^{6}]+\varepsilon$ for *r* ≤ 2^1/6^ *σ*, and zero for *r* > 2^1/6^ *σ*, where *ε* is the depth of the potential well, *σ* is the diameter of each bead, and *r* is the distance between the beads. We use the finitely extensible nonlinear elastic (FENE) spring interaction to interconnect neighboring beads, given by ${U}_{FENE}=-\frac{1}{2}k{R}_{0}^{2}\,\mathrm{ln}\,\mathrm{(1}-{r}^{2}/{R}_{0}^{2})$, where *k* is the spring constant and *R*_0_ is the maximum allowed distance between consecutive beads. We introduce the stiffness of the chain by adding an angle dependent cosine potential *U*_bend_(*θ*_*i*_) = *κ*_*b*_(1 − cos*θ*_*i*_) between successive bonds, which connect (*i* − 1)^th^ and *i* ^th^, and the *i* ^th^ and (*i* + 1)^th^ beads, where the bending rigidity *κ*_*b*_ is the interaction strength.

The physical wall is constructed by using the repulsive LJ interaction ${U}_{{\rm{LJ}}}=4\varepsilon [{(\frac{\sigma }{x})}^{9}-{(\frac{\sigma }{x})}^{3}]$, where *x* is the coordinate in the direction perpendicular to the wall. The region of space with *x* < 0 is called the *cis* side and with *x* > 0 is the *trans* side. To construct the pore, 16 beads with diameter of *σ* are placed on a circle with diameter of *d* = 3*σ*. The center of the pore is at *x* = 0 and the pore is parallel to the wall. The thickness of the pore is *σ* and the interaction between monomers and the pore particles is repulsive LJ with the same parameters as of the excluded volume interactions between the polymer beads. The external driving force, *f*, which is in the positive *x* direction, only acts to the beads that are inside the pore.

Using Langevin dynamics the equation of motion for the *i*th bead is written as $m{\ddot{r}}_{i}=-\nabla ({U}_{{\rm{LJ}}}+{U}_{{\rm{FENE}}}+$ ${U}_{{\rm{bend}}}+{U}_{{\rm{ext}}})-\eta {v}_{i}+{\xi }_{i}$. Here, *m* in the mass of each monomer, *η* is the friction coefficient of the solvent, *v*_*i*_ is the monomer velocity, and *ξ*_*i*_ is an uncorrelated random force with 〈*ξ*_*i*_(*t*)*ξ*_*j*_(*t*′)〉 = 2*ηk*_*B*_ *Tδ*_*i*,*j*_ *δ*(*t* − *t*′). By using LJ units, the mass of each bead is chosen as *m* = 1, the length is expressed in the unit of *σ*, and the unit of time is $\sigma \sqrt{(m/\varepsilon )}$. Temperature *T* is measured in units of *ε*/*k*_*B*_, and the unit of energy is *ε* = *k*_*B*_ *T*. In LJ units the parameters of the interactions potential, length, mass, spring constant, maximum allowed distance between consecutive beads, bending rigidity, and friction coefficient have been chosen as *ε* = 1, *σ* = 1, *m* = 1, *k* = 30, *R*_0_ = 1.5*σ κ*_*b*_ = 30, and *η* = 0.7, respectively, and the external driving force as *f* = 5, 10 and 20. Here, *k*_*B*_ *T* = 1.2.

In our simulations, we have used the coarse grained bead-spring model. According to the relation ${\ell }_{p}={\kappa }_{b}/({k}_{B}T)$ in 3D, with the value of *κ*_*b*_ = 30, the persistence length is ${\ell }_{p}\,=\,25$. As the persistence length of DNA is 150 bps, in our model each bead corresponds approximately to 6 bps. The mass of a bead is about 3744 amu while its size is chosen as *σ* = 2 nm, and the interaction strength is 3.39 × 10^−21^ J at room temperature (*T* = 295 K). Therefore, the time scale in LJ unit is 85.6 ps. By assuming the effective charge of 0.094 e for each unit charge^51, 52^, twelve unit charges per bead and with a force scale of 2.0 pN, an external driving force of *f* = 10 corresponds to a voltage of 200 mV across the pore.

In the beginning of the translocation process, first we fix the first bead (head of polymer chain) at the pore and equilibrate the system in the *cis* side, after which we start the actual translocation by turning on the external driving force and releasing the first bead at *t* = 0. The translocation time *τ* is defined as the time when the last bead of the chain enters to the *trans* side. It is important to note that reflective boundary conditions must not be used for the chain, but in the case the chain escapes from the pore to the *cis* side, the translocation must be re-started from a new equilibrium configuration at *t* = 0.

### Experiment

Translocation data was collected as reported in Carson *et al*.^14^ Briefly, a solid-state nanopore chip (5 × 5 mm) that contains a freestanding, 20 *μ*m × 20 *μ*m low-stress 25-nm-thick silicon nitride window was exposed to a finely focused electron beam in a transmission electron microscope (JEOL 2010F) to make a pore in the diameter range 2.5–4 nm. Then, the chip was treated with a hot piranha solution to clean the pore surface, followed by cooling and copiously rinsing with water. Assembly of the chip in a two-chamber cell, each equipped with an electrode, allows current to be measured when voltage to the electrodes is applied. The electrodes were connected to a Chimera Instruments VC100 (New York, NY), which acquires current samples at 4.19 MHz. The signal was digitally low-pass filtered at 200 kHz prior to analysis in order to reduce the capacitance noise. After evaluating the pore conductance using an electrolyte buffer (400 mM KCl, buffered to pH 7.9 using 10 mM Tris and 1 mM EDTA), DNA samples with different lengths were added to one of the chambers to concentrations in the nanomolar range, and DNA transport data was collected at 200 mV applied voltage. DNA samples used here were purchased from Thermo Scientific (Waltham, MA). After acquisition of the data, analysis was performed using MATLAB-based OpenNanopore software^53^. Mean transport times were obtained by statistical analysis of >1000 molecular transport events for each DNA length.

## Electronic supplementary material

Supplementary Info
