# Supplementary material for: Driven translocation of a semi-flexible polymer through a nanopore
Source: Sci Rep. 2017 Aug 7;7:7423. doi: 10.1038/s41598-017-07227-3 (PMC5547125; doi:10.1038/s41598-017-07227-3)
Supplement: Supplementary file 1 — Supplementary Info [file 41598_2017_7227_MOESM1_ESM.pdf]

Supplementary Information for: Driven translocation of a semi-flexible polymer through a nanopore

Jalal Sarabadani[1,*], Timo Ikonen[2], Harri Mökkönen[1], Tapio Ala-Nissila[1,3], Spencer Carson[4], and Meni Wanunu[4]

[1]Department of Applied Physics and COMP Center of Excellence, Aalto University School of Science, P.O. Box 11000, FI-00076 Aalto, Espoo, Finland
[2] VTT Technical Research Centre of Finland Ltd., P.O. Box 1000, FI-02044 VTT, Finland
[3]Department of Mathematical Sciences and Department of Physics, Loughborough University, Loughborough, Leicestershire LE11 3TU, UK
[4]Department of Physics, Northeastern University, Boston MA 02115
[*]jalal.sarabadani@eaalto.fi

ABSTRACT

This document includes Supplementary Information for: "Driven translocation of a semi-flexible polymer through a nanopore", by J. Sarabadani, T. Ikonen, H. Mökkönen, T. Ala-Nissila, S. Carson and M. Wanunu

End-to-end distance formula

We propose the following semi-analytic expression for the end-to-end distance of a semi-flexible polymer chain with contour length N and persistence length $\tilde{\ell}_p$:

$$\tilde{R}_N = \left\{ \tilde{R}_F^2 - \frac{\tilde{R}_F^4}{2a_1N^2}\left[1-\exp\left(-\frac{2a_1N^2}{\tilde{R}_F^2}\right)\right] + 2\tilde{\ell}_pN - \frac{2\tilde{\ell}_p^2}{b_1}\left[1-\exp\left(-\frac{b_1N}{\tilde{\ell}_p}\right)\right]\right\}^{\frac{1}{2}}, \quad (1)$$

where $A = 0.8$ and $\tilde{R}_F = A\tilde{\ell}_p^{\nu_p}N^{\nu}$, with $\nu_p = 1/(d+2)$ (here $d = 3$). Equation (1) correctly recovers the scaling of the fully flexible self-avoiding chain in the limit $N/\tilde{\ell}_p \gg 1$[1] as $\tilde{R}(N/\tilde{\ell}_p \gg 1) = \tilde{R}_F = A\tilde{\ell}_p^{\nu_p}N^{\nu}$. In the opposite stiff or rod-like chain limit of $N/\tilde{\ell}_p \ll 1$, Eq. (1) gives the end-to-end distance as $\tilde{R}_N = \sqrt{a_1+b_1}N$, where by setting $a_1+b_1 = 1$ (e.g. $a_1 = 0.1$ and $b_1 = 0.9$) we recover the trivial result that $\tilde{R}_N = N$. In the intermediate regime $N/\tilde{\ell}_p \sim 10^2$, the end-to-end distance is obtained from Eq. (1) as $\tilde{R}_N = 2\tilde{\ell}_pN$ which is a characteristics of the Gaussian chain.

To show the validity of the expression for the end-to-end distance, in Fig. 1 we compare results from Eq. (1) with Molecular Dynamics (MD) simulations by presenting the normalized end-to-end distance $\tilde{R}_N^2/N^{2\nu}$ as a function of the chain length N for various values of the bending rigidity $\kappa_b = 6$ (purple squares), 15 (turquoise diamonds), 30 (red circles), 60 (green upward triangles) and 120 (orange downward triangles) which correspond to $\ell_p = 5$ (purple dashed line), 12.5 (turquoise dashed-dotted line), 25 (red solid line), 50 (green dashed-dashed-dotted line) and 100 (orange dashed-dotted-dotted line), respectively. As can be seen, Eq. (1) is able to reproduce the end-to-end distance of semi-flexible polymers with different persistence lengths for a wide range of chain parameters. In particular, in the intermediate regime between the stiff rod and fully flexible self-avoiding chains Eq. (1) correctly describes the Gaussian behavior.

Trans side friction

The *trans* side friction discussed in the main article has complicated dependence on the physical parameters of the translocation process. We have extracted it numerically from the MD simulations by calculating the normalized angular cosine-correlation function $C(n) = \cos\delta_1\cos\delta_2\ldots\cos\delta_n/\cos\delta_1$ (cf. Fig. 2(b)) for each integer $\tilde{s} = n+1$. To estimate the friction on the *trans* side, we define a cut-off value n^* for the correlation function such that $C(n^*) = 1/e$. The actual contribution to the friction in given by the values of $C(i) < C(n^*)$. Then, the *trans* side friction for the given $\tilde{s}$ is written as $\eta_{\rm TS}(\tilde{s}) = \sum_{i=1}^{n^*}\cos\delta_i$. In Fig. 3(a) we show the numerically extracted *trans* side friction as a function of the translocation coordinate $\tilde{s}$ for fixed chain length of $N_0 = 64$, bending rigidity $\kappa_b = 30$ and for different values of the external driving force $f = 5, 10$ and 20. In Fig. 3(b) the same quantity as in Fig. 3(a) is presented as a function of $\tilde{s}$ but for a fixed value of $f = 20$ and different values of the bending rigidity $\kappa_b = 2.4, 6, 30$ and 60. We can identify three distinct regimes in $\tilde{\eta}_{\rm TS}(\tilde{s})$. For small $\tilde{s}/N_0$, we find that the friction grows

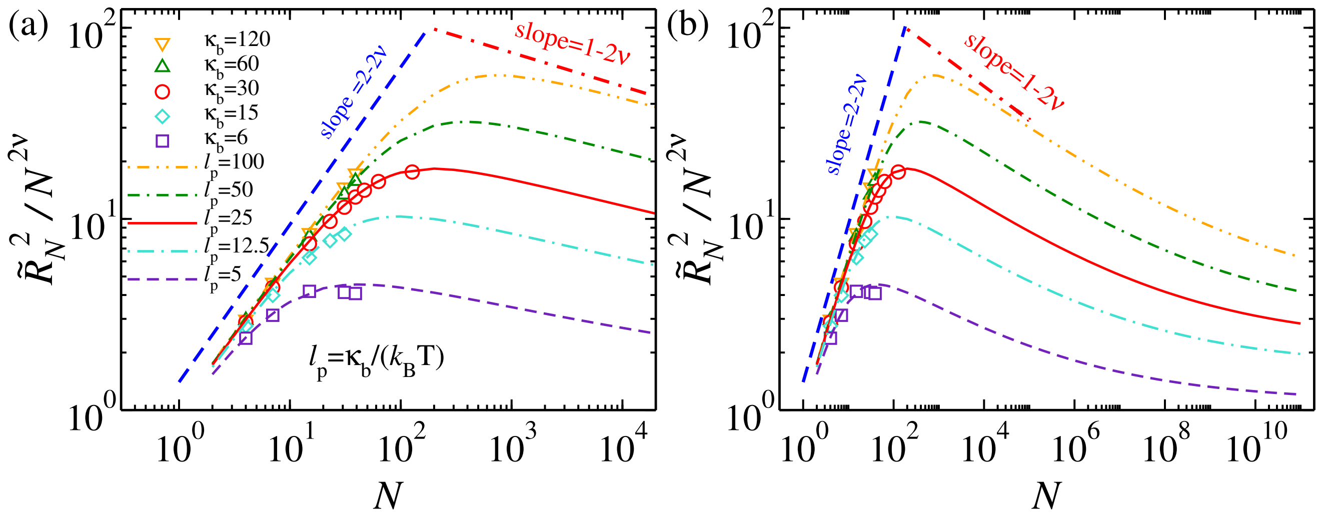

Figure 1. (a) Normalized end-to-end distance $\tilde{R}_N^2/N^{2\nu}$ as a function of the contour length of the polymer N for $k_BT = 1.2$ and various values of the bending rigidity (in the MD simulations): $\kappa_b = 6$ (purple squares), 15 (turquoise diamonds), 30 (red circles), 60 (green upward triangles) and 120 (orange downward triangles), which correspond to $\ell_p = 5$ (purple dashed line), 12.5 (turquoise dashed-dotted line), 25 (red solid line), 50 (green dashed-dashed-dotted line) and 100 (orange dashed-dotted-dotted line), respectively, according to $\ell_p = \kappa_b/(k_BT)$ in 3D. The lines are from the analytical formula of Eq.(1). (b) An extended range of N shows how the scaling of $\tilde{R}_N$ eventually crosses over to that of a self-avoiding chain at very large $N/\tilde{\ell}_p$ from the intermediate range Gaussian behavior. In the MD simulations the chain lengths are $N = 5, 8, 16, 24, 32, 40, 64$ and 128 for $\kappa_b = 30$, and $N = 5, 8, 16, 24$ and 32 for $\kappa_b = 6, 15, 60$ and 120.

proportional to the x component of the end-to-end distance $\tilde{R}_x$. After this initial stage it saturates to a constant value (for example 10.63 for $f = 10$), which from the MD simulations indicates buckling of the *trans* part of the chain. This buckling of the chain reduces the friction and we find an approximately exponential decay of the friction towards another constant value $\tilde{\eta}_{\mathrm{TS}}(N_0) \approx 5.5$ (see Fig.3). There is currently no analytic formula available for $\tilde{\eta}_{\mathrm{TS}}$.

As explained above and also in the main text of the article, there are three regimes for the trans side friction. Here, we elaborate on the physical mechanisms behind these regimes. According to our MD simulations at the early stages of the translocation process $\tilde{s}/\tilde{\ell}_p \ll 1$ the trans side chain is rod-like. Therefore, the trans side friction increases roughly linearly. At intermediate times where $\tilde{s}/\tilde{\ell}_p = \mathscr{O}(1)$, the chain has advanced far enough such that the trans side starts to bend due to fluctuations and increased friction of the solvent. In this regime the friction saturates to an intermediate value, which becomes larger for either increasing driving force (cf. Fig.3(a)) or stiffness (cf. Fig.3(b), see also Ref.[2] where similar behavior has been observed). In the late stages of translocation where $\tilde{s}/\tilde{\ell}_p \gg 1$, the trans side friction approaches its asymptotic constant value $\tilde{\eta}_{TS}(\tilde{s} \to N_0)$. As can be seen in Fig. 3(b), in the limit of fully flexible chains the asymptotic constant value is rapidly attained and can thus be incorporated in a constant, effective pore friction as we have already previously shown[3].

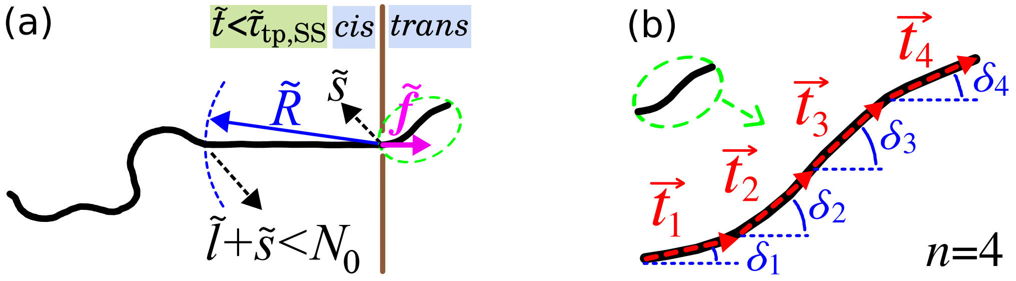

Figure 2. (a) A schematic of the translocation process in the tension propagation stage, i.e. $\tilde{t} < \tilde{t}_{\mathrm{tp,SS}}$, for the strong stretching regime. (b) A schematic representation of the chain in the *trans* side when $\tilde{s} = 5$, in the green dashed ellipsoid in the *trans* side of panel (a). The tangential vector $\vec{t}_i$ connects beads with translocation coordinates $\tilde{s}_i$ and $\tilde{s}_{i+1}$ and therefore here the number of tangential vectors on the *trans* side is $n = 4$. The angle between $\vec{t}_i$ and the direction of the external driving force $\tilde{f}$, which is $\hat{x}$, is denoted by δ_i.

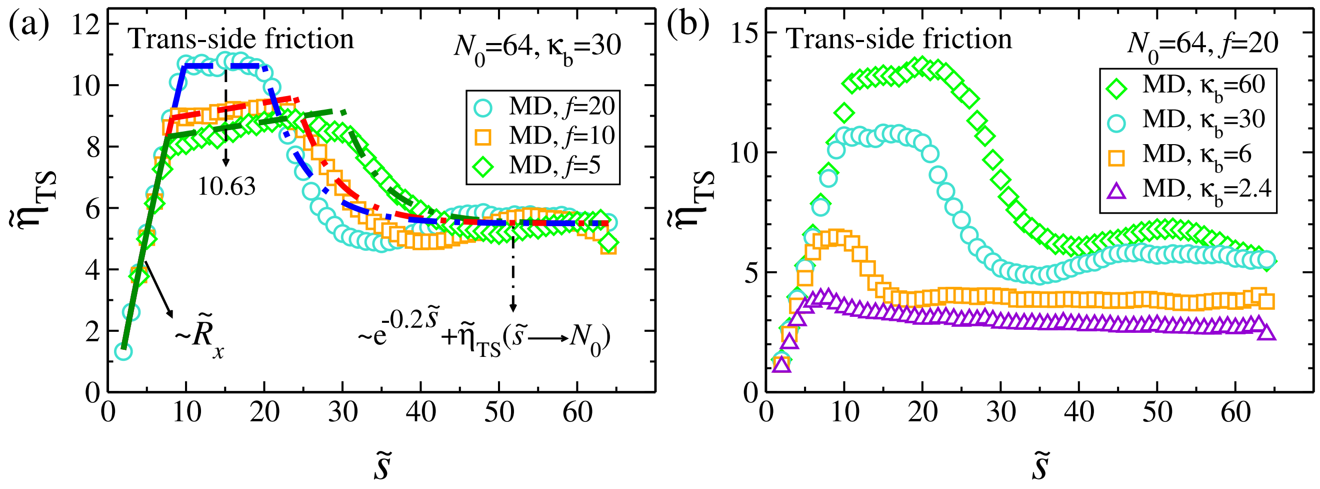

Figure 3. (a) The *trans* side friction $\tilde{\eta}_{\rm TS}$ as a function of the translocation coordinate $\tilde{s}$ for chain length $N_0 = 64$, bending rigidity coefficient $\kappa_b = 30$ and various values of the external driving force $f = 5, 10$ and 20. The green diamonds ($f = 5$), orange squares ($f = 10$) and turquoise circles ($f = 20$) are MD data. For $f = 20$, the blue solid line represents the *trans* side friction at the beginning of the translocation process, which is proportional to the x component of the end-to-end distance. The horizontal blue dashed line shows that the *trans* side friction has a constant value of ≈ 10.63 during the first buckling stage. Finally, the blue dashed-dotted line exhibits the *trans* side friction after the buckling has already occurred, demonstrating an exponential decay to the asymptotic value of the *trans* side friction, $\tilde{\eta}_{\rm TS}(\tilde{s} \to N_0)$. The green and red lines represent these approximate analytical fits for the *trans* side friction for $f = 5$ and $f = 10$, respectively. (b) $\tilde{\eta}_{\rm TS}$ as a function of $\tilde{s}$ for chain length $N_0 = 64$, external driving force $f = 20$ and various values of the bending rigidity coefficient $\kappa_b = 2.4, 6, 30$ and 60. The green diamonds ($\kappa_b = 60$), orange squares ($\kappa_b = 30$), turquoise circles ($\kappa_b = 6$) and violet triangles ($\kappa_b = 2.4$) are MD data.

Waiting time distribution

In Fig. 4 we show the waiting time distribution $w(\tilde{s})$, which is the time that each bead spends at the pore, as obtained from the MD simulations (blue triangles). The pink dashed line is the result obtained from the previous IFTP theory of Ref.[3] by assuming that the *trans* side friction is implicitly included in $\eta_{\rm p} = const.$, which is an excellent approximation for the fully flexible chains. The data clearly show that in order to have a quantitative theory, we must include $\tilde{\eta}_{\rm TS}(t)$ in Eq. (3) of the main article.

Translocation time exponent

In Fig. 5 the effective translocation time exponent α is plotted for different external driving forces $f = 5$ (green dashed line), 10 (orange solid line) and 20 (blue circles) as a function of the chain length, N_0, for fixed values of persistence length $\ell_p = 25$ and pore friction $\eta_p = 4$. As can be seen, the value of α in the very short chain limit $N_0/\tilde{\ell}_{\rm p} < 1$, and for the Gaussian regime and beyond it, does not change if the external driving force varies from 20 to 5, while for $1 < N_0/\tilde{\ell}_{\rm p} < 4$ the values of α for different values of the force $f = 5$, 10 and 20 are not the same.

Scaling of the translocation time

Following Ref.[3], to obtain an analytical form for the translocation time we assume that only the external driving force $\tilde{f}$ contributes to the total force in the BD Eq. (1) in the main article. This leads to reduction of Eq. (2) in the main article to $\tilde{\phi}(\tilde{t}) = \tilde{f}/\left[\tilde{R}(\tilde{t}) + \tilde{\eta}_{\rm p} + \tilde{\eta}_{\rm TS}\right]$. Then the total translocation time $\tilde{\tau}$ that is the sum of TP ($\tilde{\tau}_{\rm tp}$) and PP ($\tilde{\tau}_{\rm pp}$) times can be written as

$$\tilde{\tau} = \frac{1}{\tilde{f}}\left[\int_0^{N_0} \tilde{R}_N dN + \tilde{\eta}_{\rm p} N_0\right] + \tilde{\tau}_{\rm TS}, \tag{2}$$

where $\tilde{\tau}_{\rm TS} = \left[\int_0^{N_0} \tilde{\eta}_{\rm TS} dN + \int_0^{\tilde{R}_{N_0}} (\tilde{\eta}_{\rm TS,pp} - \tilde{\eta}_{\rm TS,tp}) d\tilde{R}\right]/\tilde{f}$ is the contribution from the *trans* side friction to the total translocation time. The second term in τ_{TS} is due to non-monotonic behavior of the trans-side friction $\tilde{\eta}_{\rm TS}$ in the TP and PP stages, as demonstrated in Fig. 3. Here, for the TP stage the conservation of mass is $N = \tilde{s} + \tilde{l}$ and the TP time can be obtained by integration of N from 0 to N_0, while in the PP stage the conservation of the mass is $N = \tilde{s} + \tilde{l} = N_0$ and the PP time is solved by integration of $\tilde{R}$ from $\tilde{R}_{N_0}$ to zero.

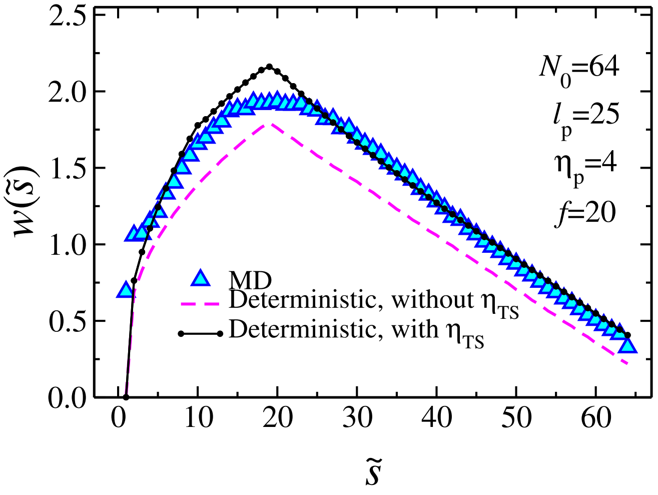

Figure 4. The waiting time distribution $w(\tilde{s})$ as a function of the translocation coordinate $\tilde{s}$. The parameters here are chain length $N_0 = 64$, persistence length $\ell_p = 25$, pore friction $\eta_{\rm p} = 4$, and the external driving force $f = 20$. The MD simulation data are presented by blue triangles. The pink dashed curve is the waiting time when the *trans* side friction is not explicitly taken into account[3]. The solid black line is the result from the IFTP theory with $\tilde{\eta}_{\rm TS}$.

In the rod-like limit the end-to-end distance of the chain is given by $\tilde{R}_N = N$. For the rod-like polymer the number of mobile monomers on the *cis* side is given by $\tilde{l} = \tilde{R}$, while on the *trans* side it is $\tilde{s}$. As the chain is stiff the TP time is much smaller than the total translocation time, i.e. $\tilde{\tau}_{\rm tp} \ll \tilde{\tau}$, therefore the TP stage can be ignored. In the PP stage, as $N = \tilde{s} + \tilde{l} = N_0$, one sets the condition $dN/d\tilde{t} = 0$ and integrates $\tilde{R}$ from $\tilde{R}_{N_0}$ to zero to obtain the PP time. Then, the translocation time becomes $\tilde{\tau} = \tilde{\tau}_{\rm pp} = \frac{1}{\tilde{f}} \int_0^{\tilde{R}_{N_0}} d\tilde{R} \big[\tilde{R} + \tilde{\eta}_{\rm p} + \tilde{\eta}_{\rm TS}(\tilde{t}) \big]$. Knowing $\tilde{\eta}_{\rm TS}(\tilde{t}) = \tilde{s} = N_0 - \tilde{l}$ together with $\tilde{l} = \tilde{R}$ yield the final scaling form as

$$\tilde{\tau} = \frac{1}{\tilde{f}} \left[\tilde{\eta}_{\rm p} N_0 + N_0^2 \right]. \qquad (3)$$

Similarly to the flexible case, the pore friction term causes a significant correction to asymptotic scaling and the corresponding effective exponents for intermediate values of N_0 will be between unity and two.

References

1. Nakanishi, H. Flory approach for polymers in the stiff limit. *J. Physique* **48**, 979–984 (1987).
2. Yang, Z.-Y. *et al.* The semiflexible polymer translocation into laterally unbounded region between two parallel flat membranes. *Polymers* **8**, 332 (2016).
3. Sarabadani, J., Ikonen, T. & Ala-Nissila, T. Iso-flux tension propagation theory of driven polymer translocation: The role of initial configurations. *J. Chem. Phys.* **141**, 214907 (2014).

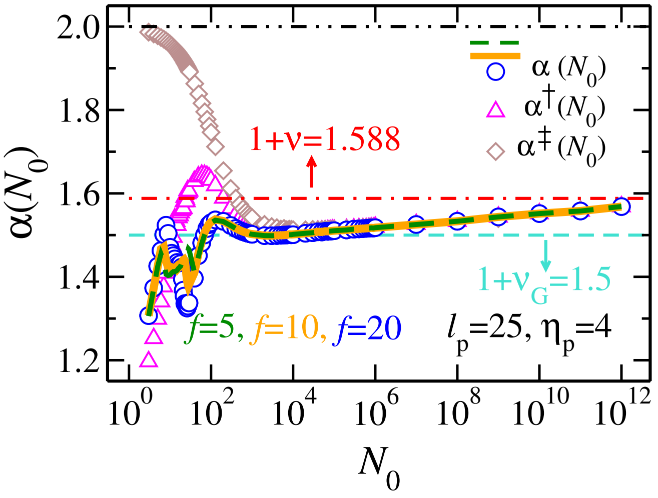

Figure 5. The effective translocation time exponent α as a function of the chain length N_0 for persistence length $\ell_p = 25$ and pore friction $\eta_\mathrm{p} = 4$, for various values of the external driving force $f = 5$ (green dashed line), 10 (orange solid line) and 20 (blue circles). The pink triangles and brown diamonds show the rescaled translocation exponents $\alpha^\dagger$ and $\alpha^\ddagger$, respectively, as a function of N_0. The horizontal black dashed-dotted-dotted, red dashed-dotted and turquoise dashed lines show the asymptotic rod-like, exculed volume and the Gaussian chain limits, respectively.
